# A mid term comparison of open wedge high tibial osteotomy vs unicompartmental knee arthroplasty for medial compartment osteoarthritis of the knee

**DOI:** 10.1186/1749-799X-5-65

**Published:** 2010-08-30

**Authors:** Ryohei Takeuchi, Yusuke Umemoto, Masato Aratake, Haruhiko Bito, Izumi Saito, Ken Kumagai, Yohei Sasaki, Yasushi Akamatsu, Hiroyuki Ishikawa, Tomihisa Koshino, Tomoyuki Saito

**Affiliations:** 1 Department of Orthopaedic Surgery, Yokohama City University, School of Medicine, 3-9 Fukuura Kanazawa-ku Yokohama city, 236-0004, Japan

## Abstract

**Background:**

The choice of surgical treatments for unicompartmental osteoarthritis (OA) of the knee is still somewhat controversial. Midterm results from cases treated using unicompartmental knee arthroplasty (UKA) or open wedge high tibial osteotomy (OWHTO) were evaluated retrospectively.

**Methods:**

Twenty-seven knees of 24 patients with varus deformities underwent OWHTO and 30 knees of 18 patients underwent UKA surgeries for the treatment of medial compartmental osteoarthritis (OA). The KSS score, FTA, range of motion and complications were evaluated before and after surgery.

**Results:**

The preoperative mean KSS scores were 49 points in the OWHTO group and 62 in the UKA group which improved postoperatively to 89 (excellent; 19 knees, good; 8 knees), and 88 (excellent; 25, good; 4, fair; 1), respectively. There was no significant difference between the OWHTO and UKA scores. Seventeen patients in the OWHTO group could sit comfortably in the formal Japanese style after surgery. The preoperative mean FTA values for the OWHTO and UKA groups were 182 degrees and 184, and at follow-up measured 169 and 170, respectively. In the UKA group, the femoral component and the polyethylene insertion in one patient was exchanged at 5 years post-surgery and revision TKAs were performed in 2 cases. In the OWHTO group, one tibial plateau fracture and one subcutaneous tissue infection were noted.

**Conclusions:**

Treatment options should be carefully considered for each OA patient in accordance with their activity levels, grade of advanced OA, age, and range of motion of the knee. OWHTO shows an improved indication for active patients with a good range of motion of the knee.

## Introduction

The correct choice of surgical treatment for unicompartment osteoarthritis (OA) of the knee is still somewhat controversial. Many patients suffering from OA have only a medial compartmental disorder and the joint surfaces of the two other compartments typically remain relatively healthy in these cases. There are a number of surgical options for treating this disorder including high tibial osteotomy (HTO), unicompartmental knee arthroplasty (UKA) and total knee arthroplasty (TKA).

HTO is a commonly used surgical procedure for the treatment of medial compartmental osteoarthritis (OA) of the knee and has wide appeal because of the preservation of the knee joint with this method relative to the use of TKA or UKA. The long-term follow-up for HTO patients has also been well documented. Billings et al. reported that 43 of 64 knees had good clinical results with an average HSS knee score of 94 points at an average of 8.5 years after HTO [1]. Koshino et al. reported long term results (15~28 years) from their analysis of 75 knees in a group of 53 patients and found that the mean KSS score improved from 37 to 87. The authors concluded that if the correct postoperative alignment is achieved, a good symptomatic improvement could be expected [1,2]. However, as a result of improvements in operative methods and in the quality of the implants used for TKA and UKA, the use of HTO has greatly decreased in recent years [3,4]. The traditional technique for treating medial compartmental OA is a lateral closed-wedge osteotomy, as previously advanced by Coventry et al., and which involves a wide dissection of the lateral soft tissue and a fibula osteotomy [5]. There are disadvantages to the use of closed-wedge HTO, however, including the long period of rehabilitation, complications associated with the surgery and the need to perform a fibular osteotomy [6].

Medial open wedge high tibial osteotomy (OWHTO) was first described by Debeyre and Artigou in 1951 [1]. More recently in 1987, Hernigou et al. reported their findings from a long-term follow-up of spontaneous osteonecrosis of the knee (SONK) patients who had undergone OWHTO [7]. There are different methodologies and implants that can be used in OWHTO and Koshino et al. have described the use of hydroxyapatite wedges in the osteotomy gap [8]. The principal advantages of OWHTO include maintenance of the bone stock, correction of the deformity close to its origin, and no requirement for a fibular osteotomy [6,9]. We have reported previously that with an early and active rehabilitation program, OA patients can walk with full weight bearing at two weeks after their OWHTO procedure [10,11]. The surgical procedures used for our patient cohort were performed using TomoFix™ as the implant (Synthes Inc., Bettlach, Switzerland), in combination with artificial bone or bone substitute, in the osteotomy gap. We have found that an optimal postoperative rehabilitation program following OWHTO in elderly cases enables these patients to walk without any support and with a full weight bearing load more quickly after their procedures.

UKA is another surgical option that has distinct advantages over other procedures including a lessening of bone resection, less dissection of soft tissues and the maintenance of nearly normal function and kinematics. Recent reports from various groups have shown excellent clinical results for UKA [3,12-14]. In addition, there are some reports comparing UKA and closed wedge HTO, but none that compare open wedge HTO (OWHTO) with other procedures such as UKA [15-17]. Rehabilitation including early full weight bearing and full range of motion training exercises, which has been a standard for UKA [14], has now been proven to be safe also for OWHTO patients [10]. The purpose of our current study was to clarify the surgical indications of OWHTO and UKA for medial compartmental osteoarthritis of the knee by retrospectively comparing midterm results for these two methods.

## Materials and methods

Our principal inclusion criterion for the OWHTO and UKA procedures investigated in this study was the diagnosis of a medial compartmental OA. In addition, a varus malalignment of the leg, isolated medial compartment osteoarthritis and active compliance with our postoperative rehabilitation program were preconditions for inclusion in our study cohorts. The range of motion of the knee had to measure at least a 20° extension to a 120° flexion. It was also important that there were no insufficiencies in the anterior or posterior cruciate ligaments, and no severe patello-femoral OA.

Twenty-eight knees of the 25 patients with varus deformities included in our study underwent an OWHTO procedure for the treatment of medial compartmental OA. These procedures took place in our hospital between 2002 and 2005. One patient with one affected knee died two years after surgery. The remaining 24 patients (18 female and 6 male), for which a total of 27 knees had OA, were further examined. The average age in this group was 67 ± 7 years (range, 54-78) at the time of surgery. The mean duration of follow-up was 61 ± 10 months. All of these patients underwent OWHTO using TomoFix [6].

**Figure 1:**
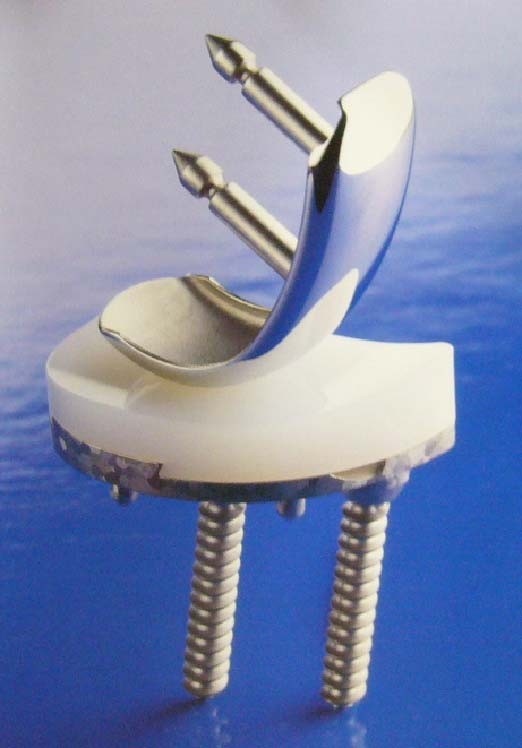
**Compartment Unicompartment-Knee**. Image of a titanium implant (Nakashima Propeller Co, Okayama, Japan). The tibial component was implanted first with or without bone cement and then firmly fixed with two screws. The femoral side is designed for surface replacement and is fixed with bone cement.

In our second study cohort, 32 knees of 20 patients underwent a UKA procedure for the treatment of medial compartmental OA. The UKA implant used in each of these cases was a Compartment Uni-Knee (Nakashima Propeller Co, Okayama, Japan) (Figure 1) [14]. These procedures took place in our hospital between 1994 and 2000. Two knees of two patients were excluded because of a revision by TKA. The remaining 30 knees of 18 patients (14 female) were thus available for further analysis. The patients averaged 77 ± 4 years of age (range, 69-86) at the time of their operations. The mean duration of follow-up was 84 ± 4 months. All patients in this study were given informed consent before surgery.

Weight bearing antero-posterior radiographs were taken in each case and all knees showed varus deformities involving medial joint-space narrowing. The progress of the OA was evaluated using a modified Ahlbäck's grading method [18]. In the OWHTO group, 11 knees were designated as Grade 2, 14 as Grade 3 and two knees showed a grade 4 deformity. Correspondingly in the UKA group, four knees were grade 2, 17 were grade 3, 8 were grade 4, and a single knee was found to be grade 5. Each of the patients presented with pain on the medial side of the knee but no history of injury or of a tibial fracture of the knee was associated with any of these cases (Table 1).

**Table 1:** Demographics data for both groups

	OWHTO	UKA
Age	67 ± 7	77 ± 4
Cases	27 knees, 24 cases	30 knees, 18 cases
Sex	man: 6, woman: 18	man: 4, woman: 14
OA Grade		
2	11 knees	4 knees
3	14	17
4	2	8
5	0	1
Follow-up period (months)	61 ± 10	84 ± 4

OWHTO; opening wedge high tibial osteotomy, UKA; unicompartmental knee arthroplasty

OA Grade; Ahlbäck's classificationion

Radiological evaluations were carried out on the femoro-tibial angle (FTA), using an AP weight bearing radiograph of a single leg, with the knee joint in extension. A weight bearing line (WBL) ratio was calculated using standing long-cassette radiographs of the lower extremities. The WBL was detected by drawing a line from the center of the femoral head to the midpoint of the proximal joint surface of the talus. The WBL ratio was then calculated as the horizontal distance from the WBL to the medial edge of the tibial plateau, divided by the width of the tibial plateau [10].

Clinical examinations of the knee joints in our cohorts consisted of objective parameters that were recorded and documented using the Orthopedic American Knee Society Knee Score (KSS) and Function Score. In this system, an excellent result is defined as a score of 85-100, 70-84 is good, 60-69 is fair and less than 60 is poor. These evaluations were carried out pre and post-surgically, and again during the post-surgery follow-up. Additional clinical findings that were assessed included range of motion and possible post-surgical complications.

In both groups of patients examined in this study, the same postoperative rehabilitation regime was performed. On the day after surgery, active and passive range of motion exercises using CPM and muscle strengthening were commenced and range of motion training with CPM was continued until two weeks after surgery. Standing exercises were also initiated as soon as possible postoperatively. One week after surgery, patients were permitted to begin partial weight bearing exercises with walker equipment and then progress to full weight bearing walking at two weeks after surgery.

The Wilcoxon signed-ranks test was used to determine statistical significance, which was assigned for P values of less than 0.05.

## Results

Post operative complications were observed in both treatment groups. In the OWHTO group, we noted an infection in one patient and a lateral tibial plateau fracture in another patient during surgery. In the case of the infection, this occurred in the subcutaneous tissue at two months after surgery in the same lower leg on which the OWHTO had been performed. The area of the infection in this patient was the distal lower leg and was quite distant from the site of the surgical wound. However, once the plate and screws had been removed, the infection eventually dissipated. A long leg cast was applied for one month in this patient and full weight bearing exercises were subsequently permitted. At the time of follow-up, the arc of motion of the knee in this individual was 150°, the KSS improved to 96 and an FTA of 169° was noted. In 11 further patients, mild pain around the pes anserinus continued until the plate was removed.

**Figure 2:**
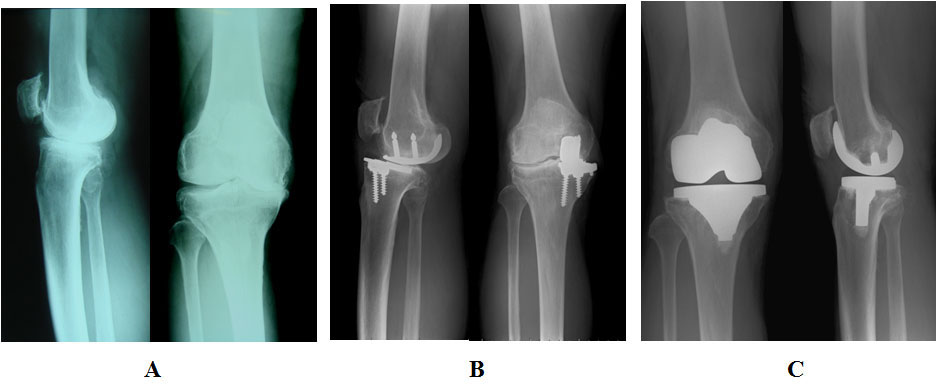
**A revision UKA case involving a 70-year-old man**. **A**. A medial compartmental grade 4 OA prior to UKA, **B**. Anterior subluxation of the tibia. Rapid wearing of the polyethylene insertion was caused by antero-posterior instability of the knee. Although a partial anterior cruciate ligament tear was observed, UKA was still recommended and performed as the initial surgery. **C**. A TKA performed two years after UKA.

In the UKA group, the femoral component and the polyethylene insertion in one patient was exchanged at five years post-surgery due to a femoral component breakage. In this same patient at two years after a second procedure, a hemoarthrosis occurred and was treated with an arthroscopic partial synovectomy. The final KSS score was 62 in this case. In two additional cases, a revision TKA was performed. In one of these patients, the first surgery was performed simultaneously with a bilateral UKA and the TKA for the right knee was undertaken at two years after the UKA procedure. This was due to a rapid wearing of the polyethylene insertion caused by an antero-posterior instability of the knee (Figure 2). Although a partial anterior cruciate ligament tear was initially observed in this same patient, UKA was still recommended at the time of the first surgery. In the other case involving a revision TKA, the femoral component was found to have broken at two years after the initial UKA procedure. There were no instances of infection found in the UKA group.

**Figure 3:**
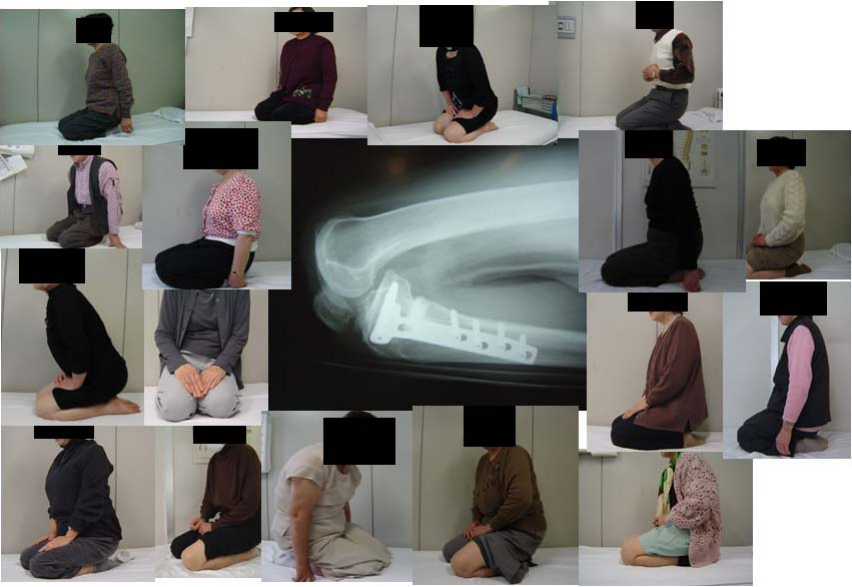
**Japanese sitting style**. Although none of the patients included in this study could sit in a Japanese style before OWHTO, 17 of the 24 patients in this group (71%) could sit comfortably in the formal Japanese style after surgery. This is an important outcome that has only been achieved thus far using OWHTO.

The average preoperative extension and flexion angle for all 27 knees in the OWHTO group and 30 knees of the UKA group, excluding the two knees that developed postoperative complications, were 3.5 ± 5.7° and 8.1 ± 5.1°, and 142 ± 10° and 123 ± 14°, respectively. After surgery, the average extension and flexion angle of both the OWHTO- and UKA-treated patients improved to 2.2 ± 3.8° and 3.0 ± 4.7° and 146 ± 5.9° and 127 ± 16°, respectively. Although none of the patients in the OWHTO group could sit in the Japanese style before surgery, 17 of these 24 patients (71%) could do so after this procedure (Figure 3).

**Figure 4:**
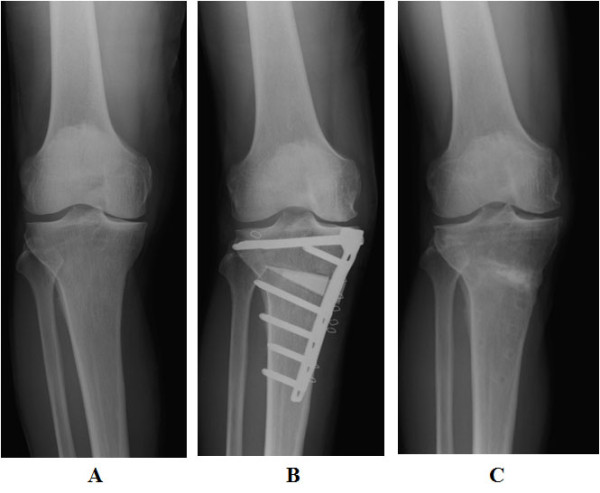
**70-year-old woman in the OWHTO group**. **A**. Prior to OWHTO, a medial compartmental grade 2 OA was diagnosed. The FTA was 182° and the KSS was 60. **B**. Two weeks after OWHTO, the FTA was corrected to 170°. **C**. Two years and 6 months after OWHTO, the FTA was maintained at 170° and there was no correction loss. This patient could subsequently sit with full flexion and the KSS improved to the excellent range.

The preoperative average KSS and Function scores in both the OWHTO and UKA groups were 49 ± 12 and 58 ± 14, and 62 ± 13 and 57 ± 8.3, respectively. After surgery, these scores in the OWHTO group improved to 89 ± 7.6 and 95 ± 7.6 (excellent, 19 knees; good, 8 knees; Figure 4) and in the UKA group to 88 ± 7.7 and 79 ± 6.8 (excellent, 25; good, 4; fair, 1), respectively. There were no significant differences found between the OWHTO and UKA groups in terms of the postoperative KSS score. However, the postoperative Function score in the OWHTO patients was significantly better than the UKA group (P < 0.01).

Prior to the OWHTO and UKA surgeries, all of the knees had a varus deformity. The preoperative average FTA values for the OWHTO and UKA groups were measured at 182 ± 2.1° (WBL ratio, 14 ± 13%) and 184 ± 3.9° (WBL ratio, 11 ± 17%), respectively. The postoperative FTA of the OWHTO group at one month post-surgery and at follow-up was 169 ± 1.4° and 170 ± 2.1° (WBL ratio, 65 ± 12%), and of the UKA group was 172 ± 3.9° and 174 ± 3.8° (WBL ratio, 57 ± 13%), respectively. There were no significant differences found between the FTA in each group at one month post-surgery or at the final follow-up. Although none of our patients in the UKA group engaged in sporting activities after surgery, five patients in the OWHTO group resumed the activities that they had engaged in prior surgery (golf, 3 patients; table tennis, 1 patient; mountain climbing, 1 patient).

## Discussion

Osteoarthritis can affect any or all three compartments of the knee but one-third of OA patients are affected in only one of these compartments [19]. The best treatment for OA in a single compartment of the knee has been the subject of much recent debate. HTO, UKA and TKA are the typical choices of surgical procedures for treating this condition. However, although there have been some comparative studies of UKA and closed wedge HTO [15,16] there are no previous reports which have compared UKA with OWHTO. Stukenborg et al. compared the clinical results in a study of 32 OA patients who underwent a closed wedge HTO and 28 such cases who were treated with UKA. These authors reported that 71% of patients in the HTO cohort and 65% in the UKA group had a KSS score of excellent or good at between 7-10 years postoperatively [17]. They further concluded that the advantages of UKA compared with closed wedge HTO include a less invasive procedure and the possibility of walking activities at an early postoperative stage. In another previous study, we demonstrated that an early weight bearing exercise program can safely enable full weight bearing at two weeks after OWHTO [10,11]. As a result of this improved postoperative rehabilitation program, we concluded that no differences between the postoperative management of UKA and OWHTO are necessary.

Ivarsson et al. have reported no significant differences between UKA and closed wedge HTO in terms of the postoperative ROM at between 6 months and 5 years after surgery [16]. On the other hand, Stukenburg et al. stated in their study that the ROM at 7-10 years postoperatively was slightly higher after closed wedge HTO compared with UKA [17]. Maintenance or restoration of a good range of knee motion is one of the most important considerations following knee surgery. However, there are currently few reports which describe postoperative range of motion in detail after a high tibial osteotomy or arthroplasty for the treatment of an OA knee [10,20]. Of note in this regard, it is very important to improve and maintain the flexion angle of the knee after surgery in Japanese cases. However, although advances in surgical techniques and prosthetic design have improved range of motion outcomes, these are currently insufficient to satisfy the cultural requirements of patients in Japan. Maximum flexion of the knee is a culturally important factor of particular note for elderly OA patients in Japan as these individuals often obliged to sit with full flexion on the floor (Japanese sitting style) as a result of local customs. It is in the range of motion of the knee, particularly the full flexion angle, that differences in the clinical outcomes between OWHTO and UKA are apparent. In this regard, Koshino et al. have reported five aspects (i.e. requirements) of sitting in a formal style on the floor: a knee flexion of more than 150°; slight internal rotation of the tibia; a plantar flexion of the ankle to 90° or more; a subtalar inversion to 45°; and no popliteal fatty mass [20]. Parratte et al. performed UKA to treat 30 cases of avascular osteonecrosis of the knee and reported that the mean KSS improved from 56 to 95 and that the mean active knee flexion angle improved from 115° to 139° [13]. This is an important outcome that has only been achieved thus far by using OWHTO.

The purpose of surgery for unicompartmental OA is to reduce pain, restore function and improve quality of life. Both OWHTO and UKA are less invasive procedures than TKA, both preserve the bone stock, and both subsequently allow for normal kinematics by retaining the anterior and posterior cruciate ligaments. Consistently, McIntosh and Hunter have stated in their previous report that the aims of hemiarthroplasty are to correct the deformity, to restore normal stability, to relieve pain, and to improve function and gait [12]. Börjesson et al. have also reported that no significant differences could be detected between the mean KSS score for UKA and closed wedge HTO at the five year follow-up stage [21]. Weale et al. have reported that the mean Function score for UKA patients was significantly better than that of a closed wedge HTO cohort [22]. In our current study, although there were no significant differences found between OWHTO and UKA procedures in terms of the postoperative KSS score, we found that the operative Function score for OWHTO was significantly better than that for UKA.

Stukenburg et al. have recently published the results of a prospective randomized analysis of a 7-10 year follow-up [17]. In this analysis, the authors reported that the revision rate after closed wedge HTO (28%) was larger than that after UKA (20%) and that this was statistically significant. Broughton et al. have also reported their findings from a comparative retrospective study of UKA and closed wedge HTO after a 5-10 year follow-up [15]. In that report, 10 out of 49 knees treated by closed wedge HTO had been revised either to TKA or arthrodesis. The authors concluded that the results with UKA were significantly better than that of closed wedge HTO. Ridgeway et al. studied the relationship between postoperative clinical results and alignment in cases treated using the UKA method [23] and concluded that the postoperative valgus alignment was important and that surgeons should avoid under correcting the FTA. In our current study, the mean FTA values for patients treated using OWHTO and UKA were ideal for postoperative valgus [24]. Saito and Koshino et al. compared the postoperative alignment and clinical results of 109 patients treated using UKA, and reported that 90 knees (82.6%) were classified as excellent under the HSS scoring system and that the FTA in most of their cases converged on a range between 170° to 175° [14]. In our current analysis, the revision rate to TKA was 6% (two knees) in the UKA group and zero in the OWHTO group. Although our follow-up period is in the mid-range, we contend that when using UKA or OWHTO only for OA knees, with strict surgical indications and post surgical alignment, excellent clinical outcomes are likely. In our OWHTO group, we noted only two surgical complications which were likely to be the result of a lack of experience in performing the OWHTO procedure.

There is considerable overlap in the surgical indications for the two treatment options reported in our present study. The important issues during the selection of an appropriate surgical treatment for unicompartmental OA include the extent of the disease in the knee, the OA grading, the preoperative alignment, the age, body weight, and activity levels of the patient, the preoperative ROM, and the effects of OA on the daily activities of the patient. OWHTO is often applied to relatively young and active patients whereas UKA has been more commonly used in elderly patients and is possible to undertake successfully even if the activity levels of the patient are low. Lotke et al. have reported in their previous review that a proximal tibial osteotomy should be considered for younger and more active patients, and have recommended that UKA and TKA should be chosen in cases that have progressed to OA of the knee [25]. The OA grading is an important consideration, and in cases of advanced OA, UKA is adopted in preference to OWHTO. Physical activities are important for many elderly patients and the mean life expectancy in Japan is greater now than ever before. Increasingly therefore, the desire for improvement in function includes an aspiration to return to sporting activity. Fisher et al. have reported that a total of 93% patients treated with Oxford unicompartmental knee arthroplasty returned to regular sporting and physical activities [26]. Their mean follow-up period was 18 months, and it is likely that further long term follow-up studies would be beneficial. However, as the knee joint is preserved by the OWHTO procedure, we would be confident in recommending a continuation of such activities for patients who had undergone this procedure.

It must be noted that as our current report is a retrospective study, the age and follow-up duration in each group do not match. In addition, a noteworthy limitation of our study design is the preoperative range of motion in the UKA and OWHTO groups. We have emphasized the importance of the flexion angle in allowing elderly patients to assume the Japanese sitting style after surgery in both groups but the preoperative flexion angle in the OWHTO group was larger than that of UKA group. There was no difference in the improvement ratio of flexion angle at the time of follow-up between both groups. We speculate that this underlies why the number of patients with severe varus deformity was greater in the UKA group than in the OWHTO group. Further randomized prospective studies will be needed in the near future to clarify the improvement in the flexion angle of the knee before and after UKA and OWHTO.

## Conclusions

Both UKA and OWHTO are useful procedures for treating cases of unicompartmental OA. The most appropriate treatment should be carefully considered for each OA patient and be based on their activity levels, the grade of advanced OA, patient age, and the range of motion of the knee. In our current study, there were no significant differences found between OWHTO and UKA in terms of the postoperative KSS score, but the postoperative Function score of OWHTO was found to be significantly better. We contend therefore that OWHTO is the more appropriate treatment for active patients who demand a good range of motion of the knee.

## Competing interests

The authors declare that they have no competing interests.

## Authors' contributions

All authors had substantial contributions to conceptions and giving final approval to the manuscript. RT, TK and TS have performed surgery, and HI, YU, MA, HB, IZ, KK, YS and YA have acted as the assistant of an operation. All datum of patients were analyzed by all authors. All authors have read and approved the final manuscript.
